# MnTE-2-PyP Suppresses Prostate Cancer Cell Growth via H_2_O_2_ Production

**DOI:** 10.3390/antiox9060490

**Published:** 2020-06-04

**Authors:** Yuxiang Zhu, Elizabeth A. Kosmacek, Arpita Chatterjee, Rebecca E. Oberley-Deegan

**Affiliations:** Department of Biochemistry and Molecular Biology, University of Nebraska Medical Center, Omaha, NE 68198, USA; yuxiang.zhu@unmc.edu (Y.Z.); elizabeth.kosmacek@unmc.edu (E.A.K.); arpita.chatterjee@unmc.edu (A.C.)

**Keywords:** MnTE-2-PyP, H_2_O_2_, prostate cancer, thiol oxidation, nuclear abnormalities

## Abstract

Prostate cancer patients are often treated with radiotherapy. MnTE-2-PyP, a superoxide dismutase (SOD) mimic, is a known radioprotector of normal tissues. Our recent work demonstrated that MnTE-2-PyP also inhibits prostate cancer progression with radiotherapy; however, the mechanisms remain unclear. In this study, we identified that MnTE-2-PyP-induced intracellular H_2_O_2_ levels are critical in inhibiting the growth of PC3 and LNCaP cells, but the increased H_2_O_2_ levels affected the two cancer cells differently. In PC3 cells, many proteins were thiol oxidized with MnTE-2-PyP treatment, including Ser/Thr protein phosphatase 1 beta catalytic subunit (PP1CB). This resulted in reduced PP1CB activity; however, overall cell cycle progression was not altered, so this is not the main mechanism of PC3 cell growth inhibition. High H_2_O_2_ levels by MnTE-2-PyP treatment induced nuclear fragmentation, which could be synergistically enhanced with radiotherapy. In LNCaP cells, thiol oxidation by MnTE-2-PyP treatment was not observed previously and, similarly to PC3 cells, there was no effect of MnTE-2-PyP treatment on cell cycle progression. However, in LNCaP cells, MnTE-2-PyP caused an increase in low RNA population and sub-G_1_ population of cells, which indicates that MnTE-2-PyP treatment may cause cellular quiescence or direct cancer cell death. The protein oxidative modifications and mitotic catastrophes caused by MnTE-2-PyP may be the major contributors to cell growth inhibition in PC3 cells, while in LNCaP cells, tumor cell quiescence or cell death appears to be major factors in MnTE-2-PyP-induced growth inhibition.

## 1. Introduction

Prostate cancer is the second most common cancer type in American men. During the past several decades, radiotherapy, chemotherapy, prostatectomy, and hormone ablation have been used to produce curative outcomes. Currently, radiotherapy is used in over 50% of prostate cancer patients [1,2]. Large amounts of reactive oxygen species (ROS) are produced by radiation, including hydroxyl radicals, superoxide and H_2_O_2_. In normal conditions, ROS act as important cell signaling molecules; however, excessive ROS damages DNA, causes lipid peroxidation and forms protein adducts [3,4]. This leads to cellular signaling pathway alterations and genetic mutation, not only to cancer cells, but also to surrounding normal cells [5]. To mitigate radiation damage of normal tissues, radioprotectors are currently being evaluated in clinical trials. One major family of radioprotectors, Mn porphyrins, have shown potency to address this problem. These porphyrins are capable of removing ROS produced by radiotherapy, thus protecting normal tissues from radiation damage.

Despite the effectiveness of radiotherapy for prostate cancer treatment, cancer cells that survive through radiotherapy become highly aggressive and resistant to future treatment, which results in patient death [6]. Targeting radioresistant prostate cancer cells has been challenging but needed for a curative outcome for late-stage prostate cancer patients. 

One potent Mn porphyrin, MnTE-2-PyP, may be able to reduce aggressive resistant prostate cancer growth [7]. MnTE-2-PyP is a manganese-based superoxide dismutase (SOD) mimic, which scavenges superoxide into H_2_O_2_ [8,9]. Many beneficial effects of MnTE-2-PyP have been reported on normal tissues. MnTE-2-PyP ameliorates radiation-induced damage during cancer-related radiotherapy [10,11]. It can also prevent cardiac arrhythmias by increasing the sarcoplasmic reticulum calcium loading capacity [12], and is efficacious in treating psoriasis and atopic dermatitis [13]. Most interestingly, recent studies showed that MnTE-2-PyP enhances tumor-bearing mice lifespan and reduces the proliferation of several prostate cancer cells lines, which indicates that MnTE-2-PyP has anti-tumor properties [14,15]. However, the exact mechanism by which MnTE-2-PyP reduces tumor growth remains elusive.

SODs are the only known family of proteins to detoxify superoxide. With different subcellular expression patterns, SODs counteract the potential damage to key macromolecules brought by superoxide [16]. Initially identified solely as antioxidant enzymes, Oberley et al. showed that SODs, especially mitochondria-localized MnSOD, are also involved in the cell cycle regulation by manipulating the H_2_O_2_ and superoxide balance [17,18,19,20]. In recent years, H_2_O_2_ has been intensely studied and its cell signaling transduction role is better understood. Many proteins carry cysteine thiol groups that are sensitive to H_2_O_2_ levels. H_2_O_2_ is able to perform reversible and irreversible modifications on these thiols, which affects protein structures, functions, and stability [21,22]. Based on the fact that MnTE-2-PyP penetrates cell membranes and accumulates in cytosolic and subcellular compartments, MnTE-2-PyP can potentially affect any macromolecular-involved pathway that is sensitive to H_2_O_2_.

Prostate cancer cells are believed to have a defective or weakened antioxidant defense system and are usually under high levels of oxidative stress [23,24]. Antioxidant enzymes, such as SODs, catalase, and glutathione peroxidases, are reported to be dysregulated or modified, resulting in loss of activity [25,26,27,28,29]. Prostate cancer cells also have higher rates of ROS generation due to their extraordinarily active mitochondria and aberrant proliferation, which produces additional ROS that may exceed the capacity of already weakened antioxidant defense systems [30]. Therefore, the anti-tumor property of MnTE-2-PyP may reside in its ability to produce excess H_2_O_2_, which cannot be handled properly in prostate cancer cells and affects regulatory cell cycle proteins or results in direct damage to key cellular structures.

We have previously shown that MnTE-2-PyP increases H_2_O_2_ levels in an aggressive prostate cancer model, PC3 cells, which causes cellular protein oxidation. Specifically, we have identified that the histone acetyltransferase, p300, is oxidized under MnTE-2-PyP treatment [31]. In combination with radiotherapy, MnTE-2-PyP significantly enhanced the survival rates of PC3 tumor bearing mice and suppressed the tumor burden as compared to MnTE-2-PyP or radiation treatment alone [14]. This indicates that MnTE-2-PyP has a synergistic effect with radiotherapy, where large amounts of ROS are generated. Based on the chemistry behind MnTE-2-PyP and poor antioxidant defense system of prostate cancer cells, we believe that MnTE-2-PyP can exaggerate the oxidative stress in prostate cancer cells and act as a prooxidant.

To test the hypothesis, we used two prostate cancer cell lines, the aggressive androgen-independent PC3 cells, and the less aggressive androgen-dependent LNCaP cells. We have recently shown that LNCaP cells have twice as much catalase activity as PC3 cells [14]. Accordingly, in this study, we found that PC3 cells had several fold higher amounts of H_2_O_2_ basally than LNCaP cells. However, MnTE-2-PyP treatment enhanced H_2_O_2_ levels about two-fold as compared to basal levels in both cell types. We found that the removal of H_2_O_2_ produced by MnTE-2-PyP resulted in the loss of MnTE-2-PyP’s ability to inhibit both PC3 and LNCaP prostate cell proliferation. We found that MnTE-2-PyP treatment inhibited cell proliferation and induced nuclear abnormalities in both PC3 (nuclear fragmentation) and LNCaP (bi-nucleation) cells, through H_2_O_2_ production. Protein oxidation and affected targets were identified, and related protein pathways were confirmed in PC3 and LNCaP cells with MnTE-2-PyP treatment. Increased pRB activation was observed in both cell lines in the presence of MnTE-2-PyP, but no significant change in cell cycle distribution was found. However, the overall number of quiescent cells, as measured by RNA production, was reduced in PC3 cells and enhanced in LNCaP cells with MnTE-2-PyP treatment. In PC3 cells, MnTE-2-PyP enhanced nuclear fragmentation, which was further enhanced by radiation and resulted in proliferation arrest and cell death. In LNCaP cells, radiation and MnTE-2-PyP enhanced the low-RNA populations and sub-G_1_ populations, indicating that MnTE-2-PyP treatment enhances cell quiescence or cell death, which contributes to LNCaP growth inhibition.

## 2. Materials and Methods

### 2.1. Cell Culture and Reagents

PC3 and LNCaP cells (human prostate cancer cell lines) were purchased from American Type Culture Collection^®^ (Manassas, VA, USA). Cells were cultured in RPMI-1640 medium (Hyclone, Logan, UT, USA) with the addition of 10% fetal bovine serum (FBS) and 1% penicillin/streptomycin in a 37 °C incubator containing 95% air and 5% CO_2_. MnTE-2-PyP (T2E) was a gift from Dr. James Crapo at National Jewish Health, Denver, CO, USA. PBS was used to dissolve MnTE-2-PyP; therefore, the same volume of PBS was added to the growth media for the control each time MnTE-2-PyP was used.

### 2.2. Animal Husbandry

Male athymic nude mice (Charles River Laboratories, Wilmington, MA, USA) were used for experiments. All mice were exposed to a 12 h light/12 h dark cycle and fed and watered ad libitum at the University of Nebraska Medical Center (UNMC, Omaha, NE, USA). All experimental protocols were reviewed and approved by the UNMC Institutional Animal Care and Use Committee (14-054-08-FC).

### 2.3. Orthotopic Implantation of PC3 Tumor Cells

The constitutive luciferase expressing PC3 cells (PC3-Luc) were purchased from Applied Biological Materials Inc. (Richmond, BC, Canada). Athymic mice were anesthetized by continuous flow of 2.5% isoflurane with oxygen using a mouse anesthesia machine. A mixture of PC3-Luc cells and Matrigel (Corning, Tewksbury, MA) was injected into the dorsal prostatic lobe (50 µL mixture containing 2 million PC3-Luc cells). The peritoneal tissues were sealed in two layers with absorbable catgut sutures (Tijuana, Mexico) and the skin was closed with wound clips (Thomas Scientific, Swedesboro, NJ, USA). Then, 0, 6, 24, and 48 h after surgery, buprenorphine (0.1 mg/kg, Reckitt Benckiser Healthcare (UK) Ltd., Hull, UK) was administrated intraperitoneally. The health condition of all mice was monitored daily, and 10 days after surgery, the wound clips were removed.

### 2.4. Radiotherapy Protocol for Mice

The tumor size was checked every week after the 5th week of orthotopic implantation by intraperitoneal D-Luciferin injection (100 mg/kg, PerkinElmer, Waltham, MA, USA) and Xenogen IVIS Spectrum bioluminescence imaging system (PerkinElmer, MA, USA). Tumor sizes of each mouse were compared by calculating the regions of interest (ROI) based on luminescence intensity. 

Mice were grouped based on the tumor size acquired from IVIS system. Tumors were CT imaged to verify size and location, then irradiated with image-guided X-rays using the Small Animal Radiation Research Platform (Xstrahl, Suwanee, GA, USA). Mice with extremely small or large tumors were excluded from the experiment. Five weeks post-surgery, mice were divided into 2 groups: PBS or MnTE-2-PyP with radiation (2 Gy per day, for five sequential days). PBS or MnTE-2-PyP (5 mg/kg) was administrated intraperitoneally 24 h before radiation and three times every week until mice were sacrificed. 

### 2.5. Tumor Harvesting and Tumor Size Measurement

Animals were sacrificed 2 weeks post-radiation treatment. The width and length of the excised tumor were measured with calipers and the volume was estimated according to the formula: [(width)^2^ × length/2]. The tumor was divided into 3 parts: one part was flash frozen and stored at −80 °C for western blot analyses; one part was fixed in 4% formalin followed by 70% ethanol, and these tissues were paraffin embedded (Tissue Science Facility, UNMC). Sections were cut and placed on slides for immunostaining. The third part of the tumor was minced in ice-cold PBS into pieces ranging from 1 to 3 mm^3^. The tumor pieces with PBS were centrifuged at 100 g for 5 min at room temperature. The supernatants were discarded, and the pelleted tumor pieces were incubated with Collagenase I (1mg/mL, Life Technologies, Eugene, OR, USA) and DNAse (100 Kunitz, Worthington, OH, USA) for 1 h at 37 °C. The digested tumor pieces were triturated about 20 times with different sized of plastic serologic pipets. Then tumor pieces were strained through a 70 µm strainer followed by 35 µm strainer (Thermo Fisher Scientific, Rochester, NY, USA). Single cells were washed with PBS and cell numbers were determined using a Coulter counter (Beckman Coulter, Indianapolis, IN, USA). The cell viability was checked by Trypan Blue assay. 

### 2.6. Cell Growth and Viability Assays

Cell growth was measured by daily cell counting for all tested conditions. Cell viability was assessed by Trypan Blue staining using a hemocytometer under bright field microscope. 

For live/dead cell imaging, cells were treated with calcein-acetoxymethyl (calcein-AM, 50 pM, Invitrogen, Carlsbad, CA, USA) and Hoechst (1 µg/mL, AnaSpec, Fremont, CA, USA) for 20 min at 37 °C in the dark. Cells were trypsinized and spun at 500 g for 3 min, and then resuspended in RPMI-1640 medium. Cell viability and nuclear morphology were observed using a LEICA CTR 6000 confocal microscope (Leica, Plymouth, MN, USA). For image quantification, average calcein-AM signal per cell was calculated.

### 2.7. Cellular H_2_O_2_ Level Measurements

To measure overall H_2_O_2_ levels, a ROS-Glo kit (Promega, Madison, WI, USA) was used as described previously [31]. Cells were treated in PBS or MnTE-2-PyP (30 µM) for 24 h. Then, cells were re-seeded in Nunclon™ 96 Flat White Plates (Thermo Fisher Scientific, Rochester, NY, USA). H_2_O_2_ levels were measured according to the manufacturer’s instructions. The luminescence signal of H_2_O_2_ was recorded by an Infinite M200 Pro Plate Reader (Tecan, Männedorf, Switzerland).

### 2.8. Altering Intracellular or Extracellular Catalase Activity

The catalase adenovirus transduction was performed as described previously [31]; cells were serum-starved in RPMI-1640 media supplemented with 2% fetal bovine serum for 6 h with corresponding multiplicity of infection (MOI) of Empty vector (ViraQuest Inc., North Liberty, IA, USA) or Ad5CMVCAT catalase adenovirus (produced by the University of Iowa Viral Vector Core). Cells were then recovered in full media with 10% fetal bovine serum for an additional 48 h.

### 2.9. Catalase Activity Measurement 

Catalase−polyethylene glycol (PEG-CAT, Sigma-Aldrich, Darmstadt, Germany) was used for scavenging extracellular H_2_O_2_. For controls, the calculated weight of polyethylene glycol (PEG, Sigma-Aldrich, Darmstadt, Germany) that corresponds to PEG-CAT was used. Different doses from 1 to 1000 U/mL were tested in cell culture for 1 or 24 h pre-treatment. 

For catalase activity staining, cells were lysed and centrifuged at 4 °C for 7 min at 12,000× *g*. Supernatants were collected and loaded onto 10% Mini-PROTEAN TGX precast gels (Bio-Rad, Hercules, CA, USA). The gel was run at 100 V for 2 h on ice and then rinsed with distilled H_2_O three times for 10 min each. The gel was incubated in 0.003% H_2_O_2_ for 10 min and rinsed twice with distilled H_2_O quickly. The staining solution (2% ferric chloride and 2% potassium ferricyanide in distilled water) was poured onto the gel immediately after rinsing, and achromatic bands were indicative of catalase activity. Gel images were inverted, and densitometry of the bands were performed using Image J (V 1.50i, Bethesda, Maryland, USA).

### 2.10. Thiol Oxidation Detection

The BIAM (N-(biotinoyl)-N’-(iodoacetyl) ethylenediamine) thiol oxidation detection methodology has been described before [31]. In brief, cell lysates were incubated with a reduced thiol-specific probe, BIAM (Life Technologies, Eugene, OR, USA) for 30 min in the dark at RT. Then, Pierce™ Streptavidin Agarose Resin (Thermo Fisher Scientific, Rockford, IL, USA) was added to each sample for 1 h at room temperature. The protein-resin complexes were washed 4 times with binding buffer and then heated at 75 °C for 10 min. The supernatant was loaded in a Bolt™ 4–12% Bis-Tri Plus gel (Invitrogen, Hercules, CA, USA) and stained by GelCode^®^ Blue Stain Reagent (Thermo Scientific, Rockford, IL, USA). 

To perform mass spectrometry analysis, protein-rich regions were cut from the gels of PBS or MnTE-2-PyP (30 µM) treatment at the same molecular weight region. An overnight in-gel trypsin digestion was performed followed by peptide clean-up using µC18 ZipTip (Millipore, Burlington, MA, USA). Each sample was resuspended in 0.1% formic acid and injected through Eksigent cHiPLC column (75 µm × 15 cm ChromXP C18-CL 3 µm 120 Å) (Eksigent Technologies, Dublin, CA, USA) onto 6600 TripleTOF (AB Sciex, Framingham, MA, USA) by typical gradient 2–60% acetonitrile in 60 min. The database search parameters were the following: database, uniprotswissprot; taxonomy, human; search method, thorough. The search results were filtered by comparing proteins identity of bands at same molecular weight in both conditions. 

### 2.11. Ser/Thr Phosphoprotein Phosphatase (PPP) Activity Measurement

Cells were treated with PBS or MnTE-2-PyP (30 µM) for 48 h. Cells were scraped in 0.5 mL protein phosphatase lysis buffer (20 mM imidazole-HCl, 2 mM EDTA, 2 mM EGTA, 1 mM benzamidine, 1 mM PMSF, and protease inhibitor, pH = 7.4). Cells were then sonicated for 3 cycles at 40% amplitude by the Model 120 Sonic Dismembrator (Thermo Fisher Scientific, Rockford, IL, USA). Cell lysates were spun at 12,000× *g* for 7 min and the supernatant was isolated. Protein concentration was measured as described above and normalized to 1 mg/mL. For PP1 activity measurement, the Ser/Thr protein phosphatase 2A (PP2A) activity were inhibited by 2 nM okadaic acid (Abcam, Cambridge, MA, USA) for 30 min. This concentration efficiently inhibits PP2A activity but not PP1 [32]. The PP1 and total PPP activity was quantified by Ser/Thr protein phosphatase Assay Kit 1 (Millipore Sigma, Billerica, MA, USA). The hydrolysis of phospho-Thr peptide was detected by Malachite green solution and measured by Infinite M200 Pro Plate Reader at 620 nm.

### 2.12. Western Blot Analysis

Cells were homogenized and protein concentrations were measured by the Bradford method. Lysed proteins of each sample were separated by a Bolt™ 4–12% Bis-Tri Plus gel and transferred onto nitrocellulose membranes using an iBlot Transfer Stack (Invitrogen, Carlsbad, CA, USA). After blocking with 5% non-reduced fat milk in TBST for 1 h, the membranes were incubated overnight at 4 °C with the following primary antibodies: PP1CB (1:500), cyclin D1 (1:10,000), phospho-cyclin D1 (Thr 286, 1:1000), pRB, phospho-pRB (Ser780, 1:1000) (Cell Signaling Technology, Danvers, MA, USA) and p16 (1:5000), p21 (1:5000) (Abcam, Cambridge, MA, USA). The secondary antibody, F (ab’) 2-goat anti-rabbit IgG (H+L) Cross-Adsorbed Secondary Antibody (1:10,000) (Invitrogen, Carlsbad, CA, USA), was used at room temperature for 1 h incubation. The blot was visualized by using Pierce™ ECL Western Blotting Substrate (Thermo Fisher Scientific, Rockford, IL, USA). Each band was quantified via ImageJ software, and the value was normalized to loading control by Ponceau (Sigma-Aldrich, Darmstadt, Germany). 

### 2.13. Cell Cycle Analysis

On the day of analysis, cells were pelleted by 500× *g* at 4 °C and then washed twice with PBS. For 4′,6-diamidino-2-phenylindole (DAPI)/Ki67 staining, cells were resuspended in 100 µL PBS, and 10 µL Ki67-FITC (Abcam, Cambridge, MA, USA) antibody was added for every 1 million cells. After 30 min incubation at room temperature in the dark, cells were washed with PBS then DAPI (1 µg/mL, Sigma-Aldrich, Darmstadt, Germany) was added. Cells were then incubated at room temperature for 15 min. In order to quantify the staining, 355/450 nm excitation/emission was used for DAPI, 488/530 nm excitation/emission was used for Ki67-FITC. The flow cytometry analysis was performed on a BD LSRII Flow Cytometer (BD Biosciences, San Jose, CA, USA). The Ki67-negative population threshold was determined based on a DAPI-only staining control. Data were analyzed using FACSDiVa analysis software (BD Biosciences, San Jose, CA, USA).

Similarly, the RNA levels were determined using pyronin (4 µg/mL, Acros Organics, Geel, Belgium) and Hoechst (10 µg/mL, BD Biosciences, San Jose, CA, USA) staining. Cells were treated with a mixture of both stains for 30 min in the dark at room temperature, and then underwent flow cytometry analysis. The 355/450 nm excitation/emission was used for Hoechest, while 488/582 nm excitation/emission was used for pyronin.

### 2.14. Nuclear Abnormality and DNA Damage Imaging

Cells were seeded in media containing PBS or MnTE-2-PyP (30 µM) for 24 h and then irradiated with 2 or 10 Gy of radiation. In some conditions, catalase transduction was applied alone or in combination with other treatments. After 72 h, cells were trypsinized and resuspended in 200 µL FBS. Cells were spun onto slides at 800 g for 3 min using a SHANDON Cytospin 3 Cytocentrifuge (Shandon, Woburn, MA, USA). Slides with cells were dried for 15 min at room temperature and then fixed in 4% paraformaldehyde for another 15 min. Slides were stored in a preservative (0.02% NaAzide in PBS) at 4 °C. PC3 tumor cells isolated from mice were processed similarly after cytospin. 

To visualize nuclear abnormalities, slides were washed twice with PBS, 10 min each. Fixed cells were mounted in 25 µL ProLong Gold antifade reagent with DAPI (Life Technologies, Eugene, OR, USA) for 30 min and then imaged using the LEICA CTR 6000 confocal microscope.

The γH2AX antibody was used as a marker for DNA double strand break in PC3 tumor sections. Formalin-fixed paraffin-embedded tissue samples were deparaffinized and heat-induced antigen retrieval was performed. Then tumor samples were permeabilized (0.1% Triton X-100 in 0.1% sodium citrate) and blocked with 2% BSA. Samples were incubated with the anti-gamma H2A.X (phospho S139) antibody (1:2000, Abcam, Cambridge, MA, USA) for overnight at 4 °C and then with goat anti-rabbit IgG (H+L) Superclonal™ Secondary Antibody, Alexa Fluor 488 for 1 h at room temperature (1:500, Thermo Fisher Scientific, Rochester, NY, USA).

### 2.15. Statistical Analysis

GraphPad Prism 8.3.1 was used for all the statistical analyses. Mean and standard deviation values from three independent experiments were used for statistical analysis for all the experiments performed. Unless otherwise described, significant differences between the groups were determined by a one-way ANOVA test followed by a post hoc Tukey’s test for multiple comparisons or a student’s t-test.

## 3. Results

### 3.1. MnTE-2-PyP Inhibits Prostate Cancer Cell Growth and Enhances H_2_O_2_ Levels

**Figure 1:**
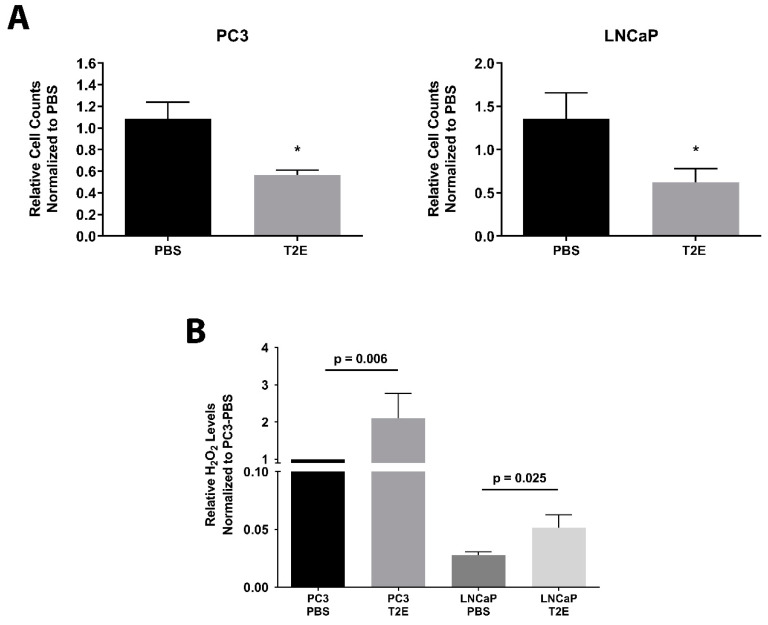
Cell growth and H_2_O_2_ levels in PC3 and LNCaP cells treated with PBS or MnTE-2-PyP treatment. (**A**) Both PC3 and LNCaP cells were treated with PBS or 30 µM MnTE-2-PyP (T2E), and cell numbers were calculated 96 h after treatment. (**B**) Intracellular H_2_O_2_ levels were measured by ROS-Glo assay. Data represent mean ± SD from at least three independent experiments. *p < 0.05 compared to PBS treatment.

Previously, we performed clonogenic experiments in both PC3 and LNCaP cells with MnTE-2-PyP and radiation treatment. MnTE-2-PyP caused a significant growth suppression in both cell lines [15]. In this study, the in vitro proliferation of PBS or MnTE-2-PyP treated prostate cancer cells was measured. PC3 cells underwent a 48.1% decrease in proliferation with MnTE-2-PyP after 4 days in culture as compared to PBS treatment (Figure 1A left panel, *p* = 0.0047), and LNCaP cells showed a 54.0% decrease in proliferation in the presence of MnTE-2-PyP (Figure 1A right panel, *p* = 0.0053). In addition, multiple time points were monitored for PC3 and LNCaP cells, we found that MnTE-2-PyP’s growth inhibition effects were not observed until day 4 and persisted for 2 weeks (data not shown). 

Significant increases (50–100%) of H_2_O_2_ levels were observed in both of PC3 and LNCaP cells with MnTE-2-PyP treatment as compared to PBS treatment; however, LNCaP cells have much lower basal H_2_O_2_ levels as compared to PC3 cells (Figure 1B). 

### 3.2. Intracellular H_2_O_2_ Balance Is Critical for Prostate Cancer Cell Growth

**Figure 2:**
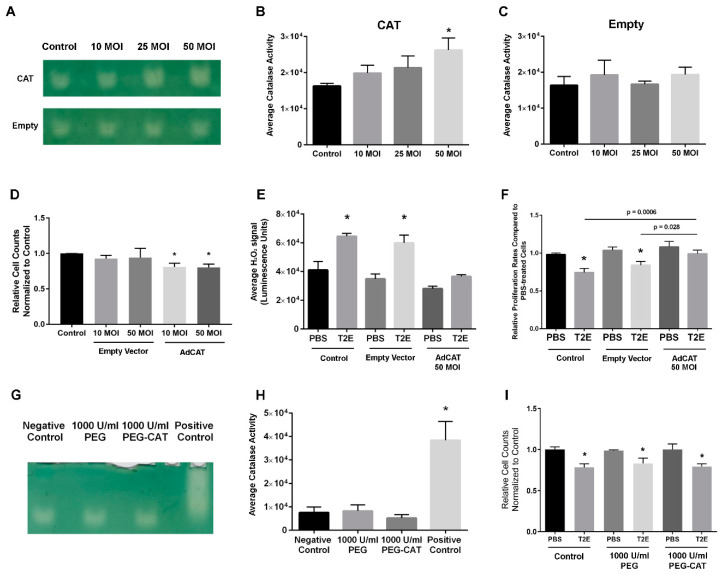
The effects of MnTE-2-PyP-induced intracellular H_2_O_2_ increase on PC3 cell proliferation. (**A**) A representative image of catalase activity in PC3 cells with different MOI catalase adenovirus (CAT)/empty vector (Empty) transduction. (**B**,**C**) Densitometry analysis of (**A**). (**D**) Relative PC3 cell counts with adenovirus transduction after 96 h. (**E**) Measurement of cellular H_2_O_2_ levels and (**F**) relative PC3 cell counts with 50 MOI catalase adenovirus/empty vector transduced after 96 h treatment of PBS or MnTE-2-PyP. (**G**) Cellular catalase activity of PC3 cells treated with PEG-catalase (1000 U/mL) or PEG (corresponding amount of 1000 U/mL PEG-catalase) for 24 h. (**H**) Densitometry analysis of (**G**). (**I**) Relative cell counts of PC3 cells after 96 h treatment of PBS or MnTE-2-PyP with PEG or PEG-catalase. All data represent mean ± SD from at least three independent experiments. *p < 0.05 compared to PBS treatment.

H_2_O_2_ can inhibit cell proliferation through redox signaling and oxidative damage. To determine whether the MnTE-2-PyP-induced H_2_O_2_ increase contributes to prostate cancer cell growth arrest, catalase adenovirus transduction and PEG-catalase were used in MnTE-2-PyP- treated PC3 cells. PC3 cells with a 50 MOI transduction of catalase adenovirus produced a significant increase in cellular catalase activity as compared to control cells (Figure 2A–C); however, catalase transduction alone caused around 20% growth inhibition in PC3 cells (Figure 2D, *p* = 0.0057 and *p* = 0.0075 for 10 and 50 MOI, respectively). PC3 cells treated with MnTE-2-PyP + catalase transduction (50 MOI) had similar H_2_O_2_ levels as compared to PBS control (Figure 2E). Correspondingly, catalase transduction rescued the growth arrest caused by MnTE-2-PyP treatment and these cells had a higher proliferation rate as compared to control or empty vector-treated cells (Figure 2F, *p* = 0.0006 and 0.028, respectively). Previous studies have shown that both intracellular and extracellular H_2_O_2_ levels can affect cell growth [33,34,35]. To identify the source responsible for the arrested PC3 cell growth, we compared the proliferation rates of PEG-catalase-treated cells with PBS or MnTE-2-PyP treatment. We found that neither 1 h (data not shown) nor 24 h pre-treatment of 1000 U/mL PEG-catalase could enhance cellular catalase activity (Figure 2G,H). As a positive control, pure PEG-catalase compound was loaded onto the gel to ensure its H_2_O_2_-scavenging activity. The PEG-catalase was unable to rescue MnTE-2-PyP-treated PC3 cells from growth inhibition (Figure 2I). These results indicate that the intracellular H_2_O_2_ balance is critical for PC3 cell growth; either the MnTE-2-PyP-induced intracellular H_2_O_2_ level increase or catalase transduction-induced H_2_O_2_ level decrease can result in cell growth arrest. 

**Figure 3:**
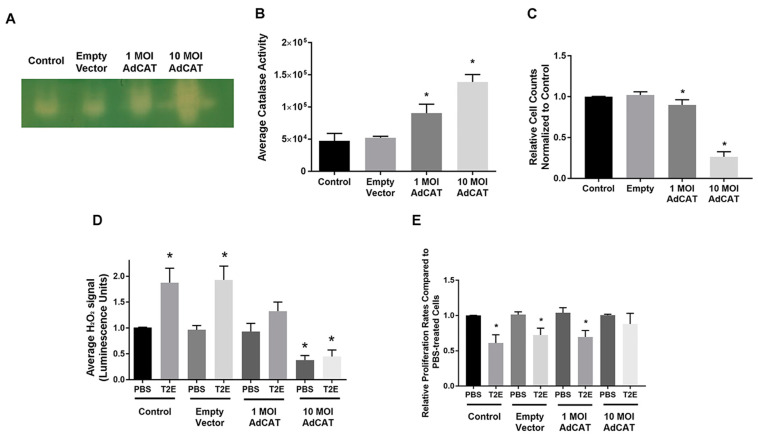
The effects of MnTE-2-PyP-induced intracellular H_2_O_2_ increase on LNCaP cell proliferation. (**A**) A representative image of catalase activity in LNCaP cells with different MOI catalase adenovirus (CAT)/empty vector (Empty) transduction. (**B**) Densitometry analysis of (**A**). (**C**) LNCaP cell growth with adenovirus transduction after 96 h. (**D**) Measurement of cellular H_2_O_2_ levels and (**E**) cell growth of LNCaP cells with 50 MOI catalase adenovirus/empty vector transduced after 96 h treatment of PBS or MnTE-2-PyP. All data represent mean ± SD from at least three independent experiments. *p < 0.05 compared to PBS treatment.

Similar experiments were performed in LNCaP cells to determine the effects of H_2_O_2_ balance on cancer growth. As compared to PC3 cells, LNCaP cells were more sensitive to catalase adenovirus transduction, and both 1 and 10 MOI catalase adenovirus transduction significantly enhanced intracellular catalase activity (Figure 3A,B, *p* = 0.0006 and 0.0001, respectively). Similarly, both 1 and 10 MOI catalase adenovirus transduction alone could significantly induce LNCaP cell growth arrest, and 10 MOI transduction greatly suppressed LNCaP cell proliferation (73.4% decrease, Figure 3C, *p* < 0.0001). Correspondingly, 10 MOI catalase transduction further decreased the H_2_O_2_ levels in both PBS and MnTE-2-PyP-treated cells to a lower extent than PBS control (Figure 3D, *p* = 0.020 and 0.035 respectively), and MnTE-2-PyP could no longer inhibit cell proliferation (Figure 3E, right panel). Although 1 MOI enhanced catalase activity, this level of transduction did not significantly decrease H_2_O_2_ levels (Figure 3D, *p* = 0.080) and could not rescue the MnTE-2-PyP-induced growth arrest (Figure 3E right panel, *p* < 0.0001). Therefore, both PC3 and LNCaP cells are sensitive to H_2_O_2_ level changes, and either an increase or decrease in H_2_O_2_ levels could alter cell proliferation; however, these two prostate cancer cell lines show different sensitivity to H_2_O_2_ levels. These data indicate that one of the main mechanisms by which MnTE-2-PyP inhibits prostate cancer is through the production of H_2_O_2_. 

### 3.3. MnTE-2-PyP Modulates Thiol Modifications in PC3 Cells

**Figure 4:**
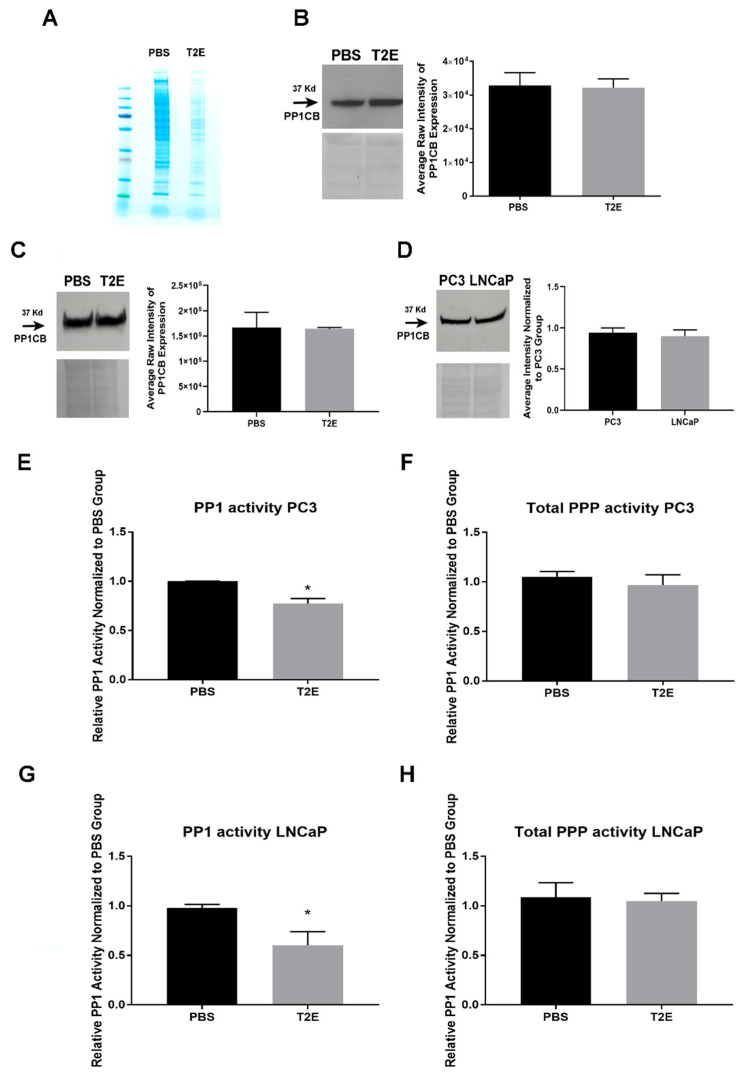
Identification of MnTE-2-PyP-induced protein oxidation and PPPs activity measurements. (**A**) A representative image of Coomassie stained gels of non-oxidized proteins in PC3 cells. Proteins with reduced thiols were isolated by N-(biotinoyl)-N’-(iodoacetyl) ethylenediamine (BIAM) probe and selected regions of Coomassie gels were cut and sent for mass spectrometry analyses. (**B**) A representative image of a PP1CB western blot with ponceau staining in PC3 cells and corresponding densitometry analysis. (**C**) A representative image of a PP1CB western blot with ponceau staining in LNCaP cells and corresponding densitometry analysis. (**D**) A representative image of a PP1CB western blot with ponceau staining in PC3 and LNCaP cells and corresponding densitometry analysis. (**E**) PP1 activity measurement in PC3 cells with PBS or MnTE-2-PyP treatment. (**F**) Total PPP activity measurement in PC3 cells with PBS or MnTE-2-PyP treatment. (**G**) PP1 activity measurement in LNCaP cells with PBS or MnTE-2-PyP treatment. (**H**) Total PPP activity measurement in LNCaP cells with PBS or MnTE-2-PyP treatment. All data represent mean ± SD from at least three independent experiments. *p < 0.05 compared to PBS treatment.

We have previously shown that MnTE-2-PyP induces protein oxidation in PC3 cells [14]. To identify which proteins were redox-modified in the presence of MnTE-2-PyP, we performed mass spectrometry analysis on cell lysates where proteins with reduced thiols were isolated by the BIAM probe. MnTE-2-PyP treatment led to a significant decrease in protein-BIAM binding in PC3 cells, indicating a decrease in proteins with reduced thiols (Figure 4A). Multiple bands in PBS and MnTE-2-PyP-treated samples with obvious differences were cut out of the gel and sent for mass spectrometry analysis. We found that Ser/Thr protein phosphatase 1 catalytic subunit (PP1CB) was detected in PBS-treated cells but not in MnTE-2-PyP-treated cells, indicating that PP1CB may be oxidized or its expression level was downregulated with MnTE-2-PyP treatment. PP1CB is one of the three most important catalytic subunits of PP1, and contains several thiol groups and one catalytic center that are highly sensitive to ROS. The oxidation of thiol groups or the Mn-Mn/Mn-Fe metal center can result in activity loss [36,37]. We measured the PP1CB protein levels in PC3 and LNCaP cells. There was no difference in PP1CB levels between PC3 and LNCaP cells (Figure 4D), and the addition of MnTE-2-PyP did not alter PP1CB expression levels (Figure 4B,C). These data suggest that PP1CB was oxidized under MnTE-2-PyP treatment. 

PP1 and PP2A contribute more than 90% of all cellular PPP activity and they are involved in various signaling pathways [38]. PP1 and PP2A can directly or indirectly control retinoblastoma protein phosphorylation (pRB), which is a key regulator of cell growth [38,39,40,41]. Under oxidative stress, PP2A is able to induce pRB dephosphorylation; meanwhile, a loss of PP1 activity can dephosphorylate pRB through the PP1-cyclin D1-pRb pathway [42,43,44]. To determine whether MnTE-2-PyP treatment altered protein phosphatases activity, we measured total PPP activity and PP1 activity by using a PP2A-specific inhibitor, okadaic acid. PP2A is often lost or inactivated in cancer through the induction of expression of endogenous inhibitors of PP2A such as CIP2A [45,46]. We found that PP1 activity was significantly decreased in both PC3 and LNCaP cells with MnTE-2-PyP treatment (Figure 4E,G), but overall PPP activity did not change in the presence of MnTE-2-PyP (Figure 4F,H), which indicates that PP2A activity may increase to compensate for PP1 activity loss.

### 3.4. MnTE-2-PyP Affects PPP-Regulated Pathways

**Figure 5:**
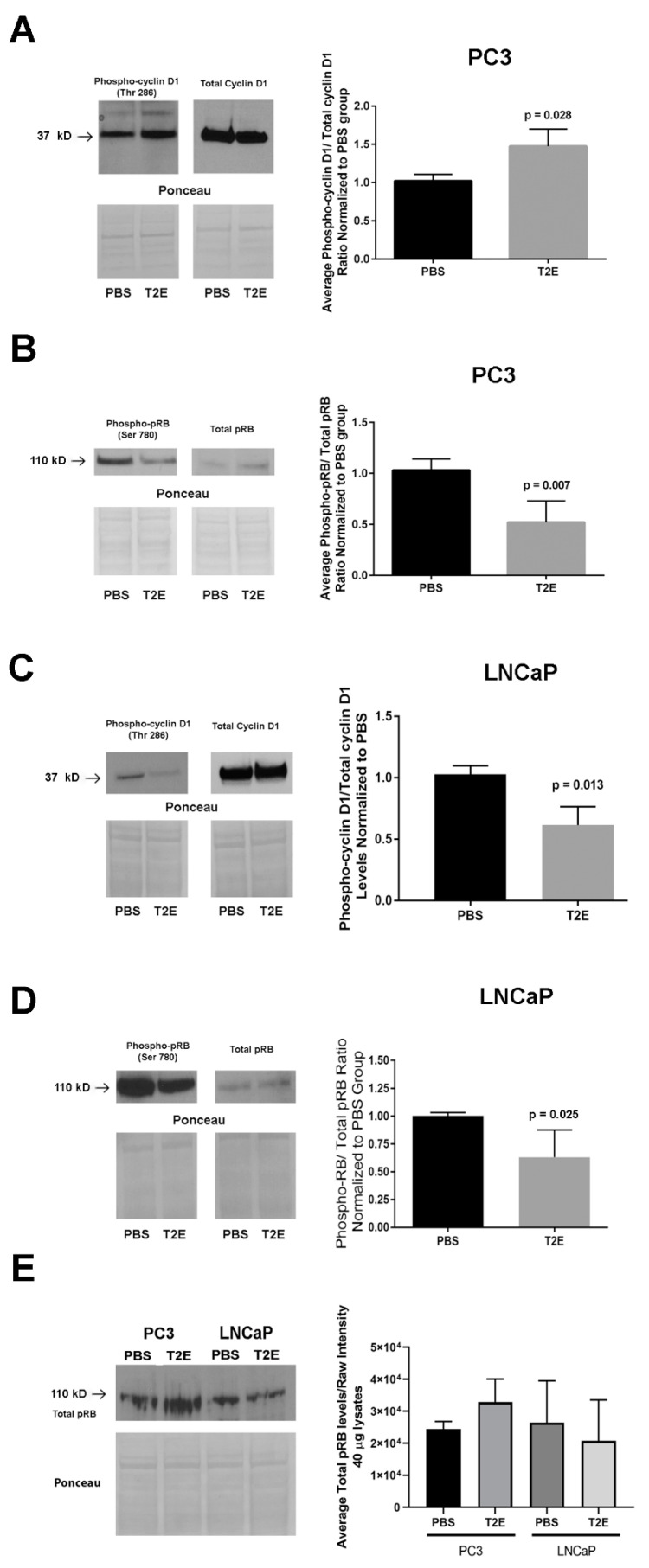

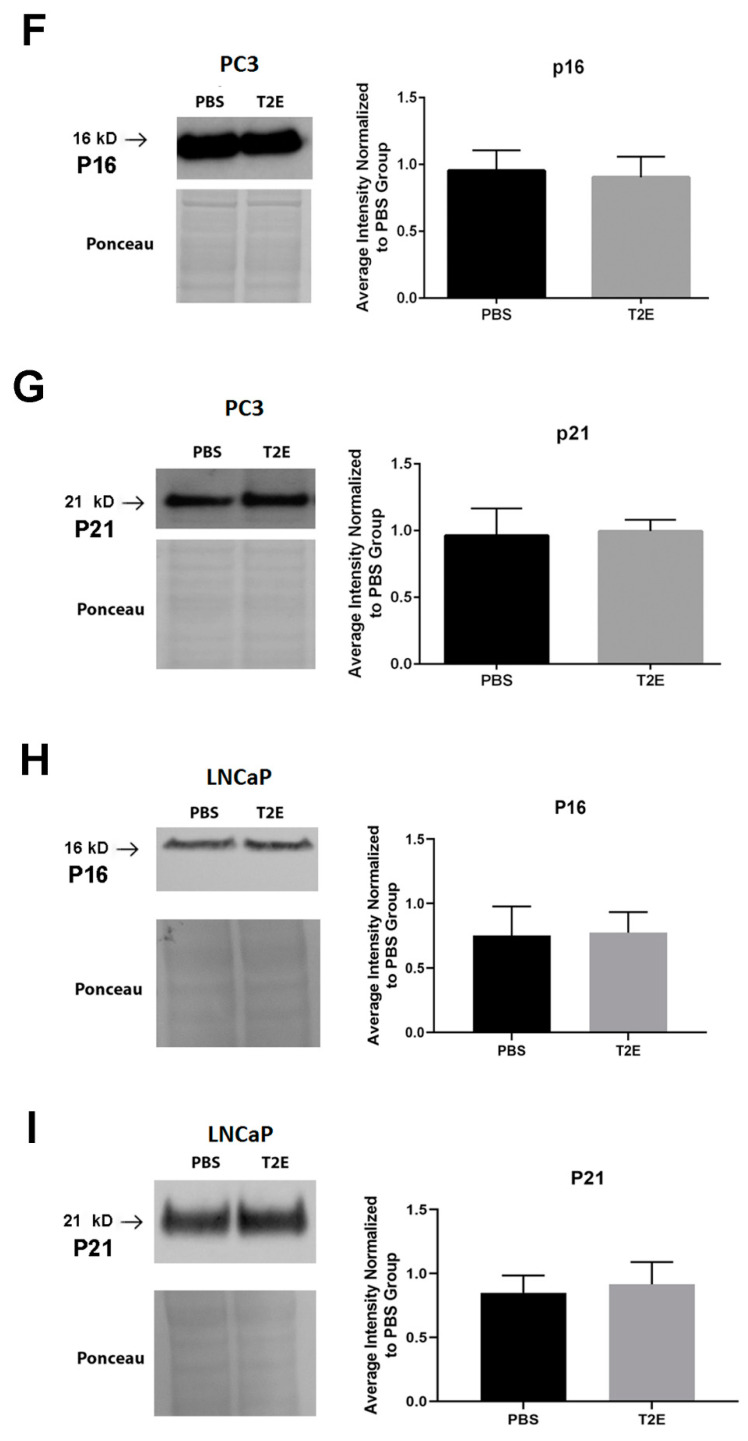
Phosphorylation levels and expression levels of PPP-regulated proteins. (**A**) Representative images of cyclin D1 and phospho-cyclin D1 (Thr 286) Western blots and ponceau staining in PC3 cells with densitometry of phosphorylation levels of cyclin D1. (**B**) Representative images of pRB and phospho-pRB (Ser-780) western blots and ponceau staining in PC3 cells with densitometry of phosphorylation levels of pRB. (**C**) Representative images of cyclin D1 and phospho-cyclin D1 (Thr 286) western blots and ponceau staining in LNCaP cells with densitometry of phosphorylation levels of cyclin D1. (**D**) Representative images of pRB and phospho-pRB (Ser-780) western blots and ponceau staining in LNCaP cells with densitometry of phosphorylation levels of pRB. (**E**) Representative images of pRB western blots and ponceau staining in both PC3 and LNCaP cells with densitometry of phosphorylation levels of pRB. (**F**,**G**) Representative images of western blots for p16 and p21 in PC3 cells, respectively, with densitometry analysis of p16 and p21. (**H**,**I**) Representative images of western blots for p16 and p21 in LNCaP cells, respectively, with densitometry analysis of p16 and p21. All data represent mean ± SD from at least three independent experiments.

We measured several downstream targets regulated by PP1 and PP2A. MnTE-2-PyP treatment enhanced cyclin D1 phosphorylation levels by 47% in PC3 cells as compared to PBS treatment (*p* = 0.028, Figure 5A). Hyperphosphorylation (Thr 286) of cyclin D1 has been shown to suppress nuclear-cytosolic transportation and alter its stability [47,48]. pRB, which is the target of cyclin D1 and suppresses excessive cell growth by inhibiting DNA replication, was hypophosphorylated in MnTE-2-PyP-treated PC3 cells (*p* = 0.007, Figure 5B); however, in LNCaP cells, both cyclin D1 and pRB were hypophosphorylated, respectively (*p* = 0.013 and 0.025, Figure 5C,D). This indicates that PP2A or other phosphatases may directly dephosphorylate pRB in LNCaP cells. We also compared pRB levels in both cell lines, but no significant difference was observed (Figure 5E). 

Several other proteins that potentially contribute to cyclin D1 hyperphosphorylation in PC3 and LNCaP cells were investigated. The expression levels of two cyclin-dependent kinase inhibitors (CKIs), p16 and p21, were measured in both PBS- and MnTE-2-PyP-treated cells, but no substantial difference in the levels of protein expression were observed (Figure 5G–I) [49,50,51]. Therefore, PP1 appears to be the major regulator of cyclin D1 phosphorylation states in MnTE-2-PyP-treated PC3 cells. 

### 3.5. MnTE-2-PyP Enhances Radiation-Induced Cell Growth Arrest in Prostate Cancer Cells 

**Figure 6:**
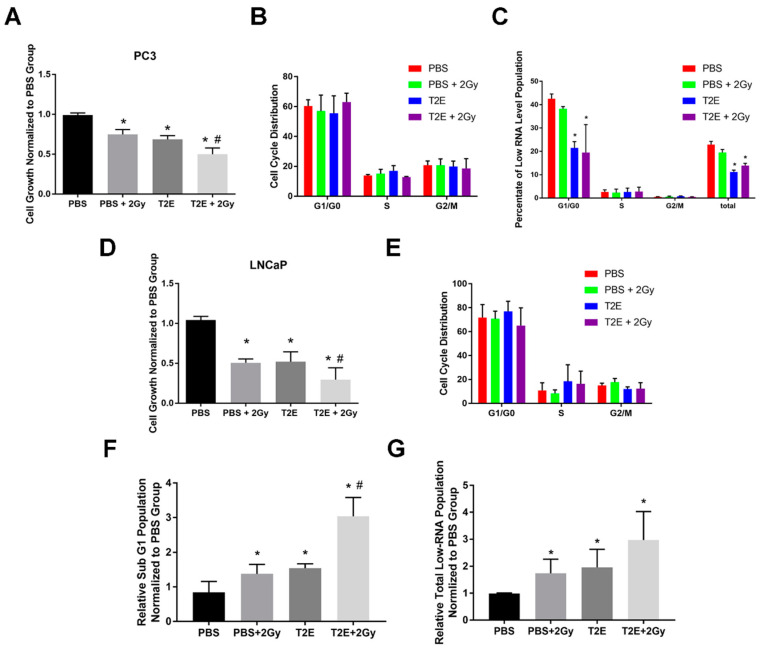
Effects of MnTE-2-PyP on cell cycle progression in combination with radiation in PC3 cells. (**A**) PC3 cells were seeded in media containing PBS or 30 µM MnTE-2-PyP. After 24 h, 2 Gy of radiation was applied to some groups. After another 72 h, cell numbers were determined. (**B**) Cell cycle analyses for PC3 cells treated with MnTE-2-PyP or radiation or the combination of the two. (**C**) Low-RNA population identified by pyronin and Hoechst staining. (**D**) LNCaP cells were seeded in media containing PBS or 30 µM MnTE-2-PyP. After 24 h, 2 Gy of radiation was applied to some groups. After another 72 h, cell numbers were determined. (**E**) Cell cycle analyses for LNCaP cells treated with MnTE-2-PyP or radiation or the combination of the two. (**F**) Measurement of sub-G_1_ population in LNCaP cells (**G**) Total low-RNA population identified in LNCaP cells by pyronin and Hoechst staining. All data represent mean ± SD from at least three independent experiments. *p < 0.05 compared to PBS treatment; #*p* < 0.05 compared to PBS + 2Gy treatment.

We have shown that MnTE-2-PyP enhanced the overall survival of animals with orthotopic implantation of PC3 cells, which was further improved when combined with radiation [14]. In support of these findings, we found that MnTE-2-PyP combined with radiation suppressed PC3 and LNCaP cells proliferation by 50% and 70%, respectively, as compared to PBS treatment after 96 h (*p* = 0.0001 and *p* < 0.0001, respectively, Figure 6A,D), and this inhibitory effect was also higher as compared to radiation or MnTE-2-PyP alone in both cell lines. 

### 3.6. MnTE-2-PyP Does Not Affect Overall Cell Cycle Distribution in Prostate Cancer Cells 

We were interested as to how MnTE-2-PyP enhanced radiation-induced cell growth inhibition. Given the alterations of pRB phosphorylation states in MnTE-2-PyP-treated PC3 and LNCaP cells, we wanted to determine whether prostate cancer cells were arrested in a particular cell cycle phase. 

In both cell lines, neither MnTE-2-PyP nor radiation exposure affected cell cycle distribution (Figure 6B,E). We measured the RNA levels in these different conditions to determine whether MnTE-2-PyP treatment led to cell quiescence. We found that MnTE-2-PyP treatment caused a decrease in low-RNA population, which means MnTE-2-PyP did not suppress PC3 cell growth through cell quiescence (Figure 6C); however, a significant increase in sub-G_1_ population and low RNA population was observed in LNCaP cells with MnTE-2-PyP or radiation treatment (Figure 6F,G), and more than 95% of the low RNA population were included in the sub-G_1_ population. Therefore, the low RNA population in sub-G_1_ population were either quiescent or dying. The above data indicate that the growth inhibition effects by MnTE-2-PyP in combination with radiation treatment are due to different mechanisms in PC3 and LNCaP cells.

PC3 and LNCaP cells have different androgen dependency. PC3 cells are androgen-independent and lack androgen receptor (AR), but LNCaP cells have functional AR signaling. We found that neither androgen deprivation treatment (ADT) nor the addition of DHT altered the effects of MnTE-2-PyP on PC3 and LNCaP cells. In both cell lines, MnTE-2-PyP inhibited cell growth similarly, regardless of ADT or DHT treatment (data not shown). Therefore, AR signaling differences do not contribute to the different responses of PC3 and LNCaP cells to MnTE-2-PyP treatment.

### 3.7. MnTE-2-PyP Treatment Induces Nuclear Abnormalities in Prostate Cancer Cells

**Figure 7:**
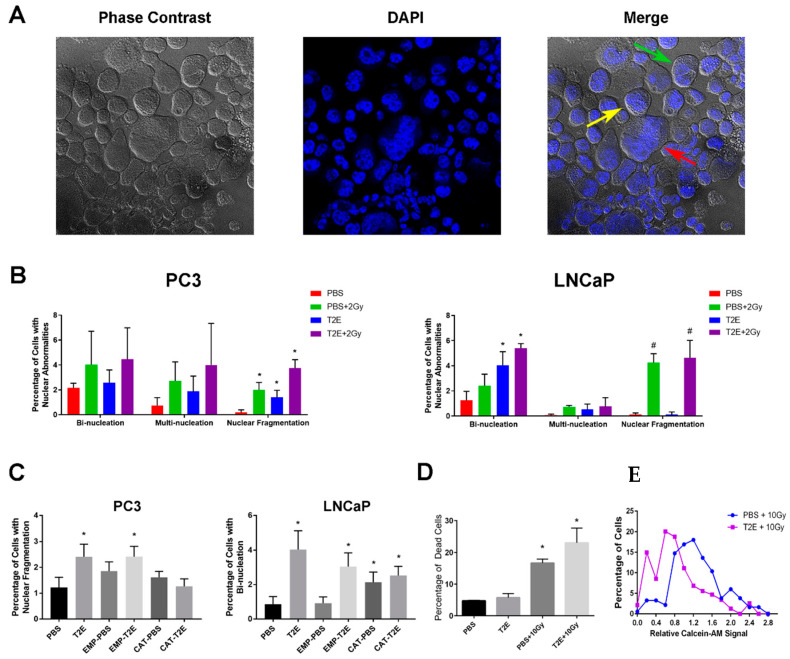
Nuclear abnormalities of prostate cancer cells treated with MnTE-2-PyP and radiation. (**A**) An example of bi-nucleated (**green arrow**), multi-nucleated (**yellow arrow**), and fragmented nucleus (**red arrow**) in PC3 cells. Cells were seeded in PBS or 30 µM MnTE-2-PyP. After 24 h, 2 or 10 Gy radiation was applied to some groups, and after 72 h, cells were fixed onto slides by cytospin. (**B**) Quantification of nuclear abnormalities in PC3 and LNCaP cells in each condition. (**C**) Quantification of nuclear abnormalities in catalase-transduced PC3 and LNCaP cells with MnTE-2-PyP + radiation treatment. (**D**) Calcein-AM staining for PC3 cells with MnTE-2-PyP and radiation treatment. Percentage of dead cells in each condition after 96 h treatment of PBS or 30 µM MnTE-2-PyP ± 10 Gy radiation. (**E**) Calcein-AM signal distribution in irradiated PC3 cells with nuclear fragmentation. All data represent mean ± SD from at least three independent experiments. *p < 0.05 compared to PBS treatment; *#p* < 0.05 compared to PBS + 10 Gy treatment.

One of the leading causes of growth inhibition by radiation is DNA damage. Therefore, we next investigated whether MnTE-2-PyP caused more radiation-induced DNA damage in vitro (Figure 7A). We found that MnTE-2-PyP or radiation or the combination of the two significantly enhanced the percentage of PC3 cells with fragmented nuclei as compared to the control (Figure 7B left panel); however, in LNCaP cells, MnTE-2-PyP alone led to bi-nucleation, and radiation alone induced nuclear fragmentation. The combination of both treatments did not further show a significant increase in either type of nuclear abnormality (Figure 7B right panel).

In order to determine whether nuclear fragmentation in PC3 cells and bi-nucleation in LNCaP cells resulted from increased H_2_O_2_ levels, both cell lines were transduced with catalase adenovirus. We found that in PC3 cells, the nuclear fragmentation caused by MnTE-2-PyP treatment could be rescued by catalase adenovirus transduction (Figure 7C, left panel). However, in LNCaP cells, catalase transduction itself caused a similar level of bi-nucleation and did not rescue the effects caused by MnTE-2-PyP (Figure 7C, right panel). Therefore, we cannot determine whether H_2_O_2_ produced by MnTE-2-PyP directly caused bi-nucleation in LNCaP cells. However, these data indicate that H_2_O_2_ balance is involved in PC3 and LNCaP cell nuclear abnormalities.

To investigate whether MnTE-2-PyP potentially induces tumor cell death through induction of DNA damage, we used calcein-AM staining on PC3 cells with PBS/MnTE-2-PyP + radiation treatment. Calcein-AM is a non-fluorescent probe that permeates into cells where it is cleaved by intracellular esterases to produce stable fluorescent signals in living cells. Since 2 Gy radiation did not yield a sufficient number of cells with nuclear abnormalities and cell death for statistical analysis, we increased the radiation dose to 10 Gy, which greatly enhanced nuclear fragmentation (60–70%) and cell death population with or without MnTE-2-PyP treatment (Figure 7D left panel). The calcein-AM staining was reduced in MnTE-2-PyP-treated samples ± radiation as compared to PBS-treated samples. Specifically, for PC3 cells with nuclear fragmentation, MnTE-2-PyP significantly enhanced the low-calcein-AM population as compared to control. This indicates that the MnTE-2-PyP + radiation can increase nuclear fragmentation, which leads to cell death or severe membrane damage (Figure 7D right panel).

### 3.8. MnTE-2-PyP treatment induces nuclear abnormalities in PC3 tumors in vivo

**Figure 8:**
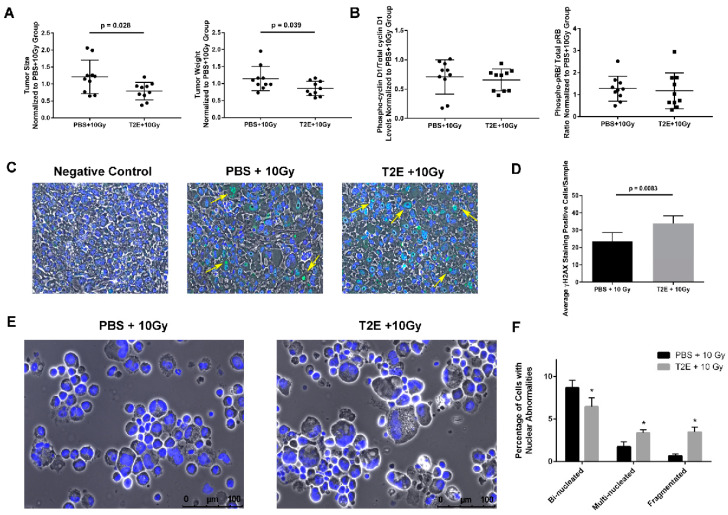
Effects of MnTE-2-PyP with radiation in PC3 tumors in vivo. (**A**) Left panel: In vivo PC3 tumor size; right panel: In vivo PC3 tumor weight after PBS or MnTE-2-PyP + 5 days x 2Gy/day treatment. (**B**) Left panel: phosphorylation level of cyclin D1 in PC3 tumor samples; right panel: phosphorylation level of pRB in PC3 tumor samples. (**C**) Representative images of γH2AX positive cells in each condition. Each arrow indicates a single cell with γH2AX positive staining, part of the positive cells were shown. (**D**) Quantification of γH2AX-positive cells in PC3 tumor sections. Each quantified image had a similar cell density. (**E**) Representative images of nuclear abnormalities in PC3 tumor samples with PBS or MnTE-2-PyP + 10 Gy radiation treatment. (**F**) Quantification of (**E**), around 1000 cells isolated from each animal’s tumor were included in the analysis. For animal experiments, *n* = 10 for both PBS + 10 Gy and MnTE-2-PyP + 10 Gy. *p < 0.05 compared to PBS + 10 Gy treatment.

To investigate the effects of MnTE-2-PyP with radiotherapy in vivo, the PC3 tumor cells were injected orthotopically into the prostate of athymic nude mice. MnTE-2-PyP in combination with radiation (2 Gy × 5 days) significantly decreased the tumor size and weight as compared to PBS + radiation treatment (Figure 8A). We measured the phosphorylation levels of cyclin D1 and pRB in the PC3 tumor cells, but there was no significant difference between PBS + radiation and MnTE-2-PyP + radiation groups (Figure 8B). We also investigated the DNA damage and nuclear abnormalities in the PC3 tumor cells. There was a significant increase in γH2AX staining in MnTE-2-PyP + radiation treatment as compared to radiation alone (Figure 8C,D), indicating there is more DNA damage in PC3 tumor cells treated with MnTE-2-PyP. Correspondingly, both multi-nucleation and nuclear fragmentation increased, with a decrease in bi-nucleation, in MnTE-2-PyP + radiation group as compared to PBS + radiation group. These above results indicate that in an in vivo PC3 tumor model, MnTE-2-PyP + radiation treatment causes more DNA damage and nuclear abnormalities, which could be partially responsible for tumor growth arrest, rather than pRB activation.

## 4. Discussion

The role that MnTE-2-PyP plays in normal tissue protection during radiotherapy has been broadly documented [8,11,52]. Our recent work indicated that MnTE-2-PyP itself also has anti-tumor properties [14]. In this study, we show that both PC3 and LNCaP cells were sensitive to H_2_O_2_ balance. MnTE-2-PyP treatment in both cell lines caused growth inhibition, pRB activation, and nuclear abnormalities in vitro, but displayed different working pathways. In vivo, MnTE-2-PyP enhanced radiation therapy and produced more nuclear fragmentation, but did not activate pRB.

Initially recognized as simple damaging molecules, ROS are now known to play important roles in cell signaling. During the past several decades, numerous publications suggest that using either antioxidants or prooxidants can inhibit cancer growth [53,54,55,56,57]. Interestingly, we found that in PC3 and LNCaP cells, either increasing H_2_O_2_ levels by MnTE-2-PyP treatment or decreasing H_2_O_2_ levels by catalase transduction ultimately led to cell growth inhibition. Moreover, catalase transduction neutralized MnTE-2-PyP’s effects on cell growth in both PC3 and LNCaP cells. By using the PEG-catalase compound that only scavenges extracellular H_2_O_2_, we demonstrated that intracellular H_2_O_2_ is the determinant factor of prostate cancer cell growth inhibition. PEG-catalase is commonly used as an alternative way to scavenge both intracellular and extracellular H_2_O_2_ and its effects on cell growth have been widely studied [58,59,60]; however, it appears that PEG-catalase (1000U/mL, 24 h treatment) did not efficiently accumulate inside the cell and exert any intracellular effects.

Thiol-specific probes allowed us to further understand individual protein modifications [61,62,63,64]. Based on the BIAM assay results, we identified that the protein, PP1CB, was oxidized due to MnTE-2-PyP treatment and the PP1-regulated pathway cyclin D1-pRB was affected in PC3 cells. PP1 has been reported to be prone to free thiols oxidation and that the metal catalytic centers are highly redox-sensitive [36,37,65]. We observed a significant decrease in PP1 activity in both PC3 and LNCaP cells when MnTE-2-PyP was used, but the overall PPP activity did not change. Based on the results that pRB was hypophosphorylated in both cell lines but the upstream protein cyclin D1 was oppositely regulated in these two cell lines, pRB hypophosphorylation may be due to increased PP2A to compensate for PP1 activity loss [44,66]. However, we did not observe a significant change in cell cycle distribution in either cell line with MnTE-2-PyP treatment (Figure 6B,E), which indicates that pRB activation is unlikely to be the major factor for MnTE-2-PyP-induced cell growth arrest. RNA measurements provided us with more insights in LNCaP cells, where low RNA populations in G_1_/G_0_ and G_2_/M phases and additional sub-G_1_ populations were observed. These data suggest that MnTE-2-PyP and/or radiation resulted in cell quiescence or cell death, which could partially explain the decreased cell proliferation [67].

Both MnTE-2-PyP treatment and radiation enhanced total nuclear abnormalities, specifically, nuclear fragmentation in PC3 cells. As compared to MnTE-2-PyP treatment alone, where no additional cell death was observed, MnTE-2-PyP combined with radiation significantly increased the percentage of dead cells (10 Gy, Figure 7D). The same experiments performed in LNCaP cells produced different results. MnTE-2-PyP induced bi-nucleation but radiation led to nuclear fragmentation, and the combination of both treatments did not significantly enhance either type of abnormality. It is possible that the difference between PC3 and LNCaP cells is due to the differences in basal H_2_O_2_ levels, where higher H_2_O_2_ levels in PC3 were more likely to exceed the tolerance of redox balance in PC3 cells and possess synergistic effects with additional ROS produced by radiation. Moreover, in vivo, PC3 tumors treated with 2 Gy/day for 5 days + MnTE-2-PyP displayed enhanced DNA damage as compared to radiotherapy alone as evidenced by γH2AX staining. The nuclear morphology analyses confirmed that MnTE-2-PyP + radiation induced more nuclear abnormalities, including multi-nucleation and nuclear fragmentation, but the bi-nucleation was decreased. This suggests that MnTE-2-PyP may convert bi-nucleation into more genomically unstable states in PC3 tumors during radiotherapy. These above data further demonstrated that MnTE-2-PyP has synergistic effects by enhancing nuclear fragmentation with radiation in PC3 cells and this could be one major mechanism to explain the improved radiotherapy, especially in more advanced prostate cancer cells.

The BIAM assay (Figure 4A) suggests that there are many more proteins affected by MnTE-2-PyP through oxidative reversible modifications in PC3 cells. These unknown targets could also potentially contribute to the cell growth inhibition and radio-sensitization effects in MnTE-2-PyP treated PC3 cells, which will be investigated in the future.

Although it remains unclear which exact pathways are affected by MnTE-2-PyP treatment that are most responsible for cell growth inhibition, based on the fact that H_2_O_2_ levels are increased and growth inhibition occurred in all prostate cancer cells tested, it is reasonable to propose that there is a link between prostate cancer growth and MnTE-2-PyP-induced H_2_O_2_ imbalance. Interestingly, based on the different responses of PC3 and LNCaP cells to MnTE-2-PyP treatment, which include RNA levels, nuclear abnormalities, and protein oxidation, it seems that MnTE-2-PyP triggers more severe oxidative stress in PC3 cells as compared to LNCaP cells. We have previously investigated the catalase levels in these two cells and we found that LNCaP cells have a two-fold higher catalase levels and activity as compared to PC3 cells [14]. In combination with the basal H_2_O_2_ levels in Figure 1B, it is possible that LNCaP cells have more reserved antioxidant defense capacity to MnTE-2-PyP-induced H_2_O_2_ than PC3 cells.

Our work showed that the MnTE-2-PyP can not only suppress prostate cancer growth, but also enhance radiotherapy efficacy. Although traditional prostate cancer therapies including radiotherapy have very high survival rates (>95%), the treatment for late-stage prostate cancer is still lacking. In this study, MnTE-2-PyP generates nuclear abnormalities and oxidative damage in the aggressive PC3 cells. Similar to the strategy in radiotherapy or chemotherapy where DNA damage is induced to suppress cancer cell growth, MnTE-2-PyP could work with DNA repair inhibitors, such as poly-ADP-ribose polymerase (PARP) inhibitor, to enhance therapeutic efficacy. In addition, the induction of cell quiescence and/or cell death in LNCaP cells indicate that MnTE-2-PyP could potentially target quiescent cancer stem cells. Therefore, combining MnTE-2-PyP with other therapies with or without radiotherapy may achieve better clinical outcomes.

## 5. Conclusions

In summary, we have shown that MnTE-2-PyP increases H_2_O_2_ levels in prostate cancer cells in both PC3 and LNCaP cells and this results in decreased proliferation. This reduction in proliferation is not due to changes in cell cycle progression. Instead, our data show that PC3 and LNCaP are inhibited by MnTE-2-PyP through different mechanisms. For PC3 cells, nuclear fragmentation and protein oxidation are likely the reasons for MnTE-2-PyP growth inhibition, which can be further enhanced by radiation. MnTE-2-PyP treatment in LNCaP cells causes cell quiescence or cell death, which likely contributes to reduced cancer cell numbers.
